# Supplementary material for: A Likelihood Approach for Real-Time Calibration of Stochastic Compartmental Epidemic Models
Source: PLoS Comput Biol. 2017 Jan 17;13(1):e1005257. doi: 10.1371/journal.pcbi.1005257 (PMC5240920; doi:10.1371/journal.pcbi.1005257)
Supplement: S1 File — (TAR.GZ) [file pcbi.1005257.s014.tar.gz › HSPH_Online-SI-Revision/EnKF07/n100-mild/n100-mild_EnKF2_table0.pdf]

1	newly 3 weeks spec	2866.	1054.7	{0., 973.}	107.776	1.56653	newly 3 weeks cumul	9776.	4712.15	{99., 2990.}	111.168	1.06933	newly 5 weeks spec	1628.	534.82	{0., 652.}	103.787	1.80823	newly 5 weeks cumul	13 712.	5990.03	{99., 4924.}	108.528	1.12914	newly 52 weeks cumul	16 805.	7744.54	{99., 11 219.}	105.755	1.13454
2	newly 3 weeks spec	3141.	640.95	{26., 1791.}	87.0764	1.10066	newly 3 weeks cumul	11 118.	2826.2	{249., 5543.}	85.2015	0.952259	newly 5 weeks spec	1875.	370.81	{11., 969.}	87.5963	1.28381	newly 5 weeks cumul	15 610.	3691.96	{374., 8901.}	84.147	0.983038	newly 51 weeks cumul	19 660.	5359.73	{431., 15 104.}	83.2095	0.965862
3	newly 3 weeks spec	2615.	1187.29	{0., 1688.}	89.8734	1.36138	newly 3 weeks cumul	10 083.	12 447.1	{3219., 11 823.}	113.353	0.416385	newly 5 weeks spec	1275.	193.65	{0., 322.}	86.862	1.55879	newly 5 weeks cumul	13 206.	13 112.8	{3443., 12 957.}	103.285	0.474439	newly 54 weeks cumul	16 122.	13 275.9	{3486., 13 257.}	99.0089	0.536397
4	newly 3 weeks spec	3002.	823.26	{59., 2542.}	79.7655	0.997219	newly 3 weeks cumul	14 624.	5484.77	{803., 11 387.}	81.9388	0.791498	newly 5 weeks spec	1149.	332.36	{3., 689.}	84.2193	1.25318	newly 5 weeks cumul	17 569.	6318.72	{927., 13 896.}	80.8421	0.794868	newly 58 weeks cumul	18 847.	7201.25	{1034., 16 819.}	79.9976	0.769495
5	newly 3 weeks spec	2761.	1223.02	{0., 2299.}	88.0507	1.2735	newly 3 weeks cumul	10 134.	10 583.1	{1859., 14 243.}	99.3603	0.420438	newly 5 weeks spec	1641.	216.51	{0., 532.}	87.3376	1.62708	newly 5 weeks cumul	14 065.	11 304.6	{2214., 15 995.}	90.7299	0.490413	newly 54 weeks cumul	17 712.	11 511.7	{2426., 16 446.}	87.5058	0.556254
6	newly 3 weeks spec	2631.	772.22	{0., 386.}	111.28	2.29077	newly 3 weeks cumul	10 483.	7618.53	{153., 23 300.}	107.706	0.870418	newly 5 weeks spec	1399.	384.78	{0., 198.}	110.435	2.59561	newly 5 weeks cumul	13 754.	8554.29	{192., 23 300.}	101.949	0.90729	newly 52 weeks cumul	16 963.	9403.46	{208., 23 300.}	99.0087	0.941042
7	newly 3 weeks spec	3036.	1260.21	{132., 1729.}	94.9904	0.859316	newly 3 weeks cumul	13 981.	6297.87	{1507., 6329.}	98.2242	0.804985	newly 5 weeks spec	924.	456.18	{15., 1153.}	81.6407	0.778364	newly 5 weeks cumul	16 613.	7476.27	{1811., 8766.}	95.5664	0.78079	newly 57 weeks cumul	17 582.	9621.48	{1992., 17 781.}	94.8668	0.711656
8	newly 3 weeks spec	2036.	931.29	{108., 631.}	109.429	0.984747	newly 3 weeks cumul	8038.	2953.67	{622., 2203.}	93.0895	0.860926	newly 5 weeks spec	1057.	382.24	{16., 328.}	100.342	1.14781	newly 5 weeks cumul	10 545.	4035.32	{820., 3144.}	95.0968	0.87209	newly 51 weeks cumul	12 212.	4826.41	{937., 5651.}	90.163	0.825326
9	newly 3 weeks spec	3348.	2373.12	{44., 9767.}	119.957	1.02657	newly 3 weeks cumul	14 156.	12 620.7	{575., 25 089.}	111.188	0.699008	newly 5 weeks spec	1368.	667.87	{3., 902.}	107.007	1.44266	newly 5 weeks cumul	17 721.	14 493.3	{617., 44 778.}	110.7	0.749133	newly 56 weeks cumul	20 118.	15 678.4	{619., 64 911.}	109.914	0.777625
10	newly 3 weeks spec	2716.	2283.31	{15., 6995.}	141.439	1.32134	newly 3 weeks cumul	9804.	5630.61	{94., 17 426.}	112.772	1.19769	newly 5 weeks spec	1557.	1392.75	{3., 6036.}	136.256	1.41293	newly 5 weeks cumul	13 469.	9109.05	{138., 30 226.}	121.617	1.19174	newly 50 weeks cumul	16 876.	20 324.1	{187., 70 271.}	143.844	1.05922
11	newly 3 weeks spec	3997.	1168.25	{0., 2197.}	94.1419	1.71449	newly 3 weeks cumul	15 533.	10 780.2	{1904., 15 896.}	98.0981	0.606242	newly 5 weeks spec	1963.	264.66	{0., 343.}	96.1355	1.97061	newly 5 weeks cumul	20 137.	11 599.8	{1904., 17 246.}	95.602	0.675768	newly 54 weeks cumul	22 930.	11 856.1	{1904., 17 493.}	94.4119	0.715234
12	newly 3 weeks spec	3071.	1683.22	{19., 1759.}	115.659	1.18227	newly 3 weeks cumul	10 922.	4225.34	{255., 7906.}	99.8193	1.07148	newly 5 weeks spec	2036.	988.45	{3., 2073.}	94.359	1.30206	newly 5 weeks cumul	15 499.	6623.81	{290., 11 659.}	100.679	1.05996	newly 50 weeks cumul	21 968.	18 476.2	{346., 84 687.}	112.377	0.946715
13	newly 3 weeks spec	3153.	952.9	{0., 1591.}	89.5065	1.89602	newly 3 weeks cumul	12 554.	14 186.3	{3242., 22 394.}	91.4567	0.36523	newly 5 weeks spec	1478.	460.09	{0., 924.}	96.366	2.41118	newly 5 weeks cumul	16 279.	15 262.8	{3838., 31 353.}	85.2984	0.397306	newly 55 weeks cumul	18 456.	17 087.2	{4416., 32 835.}	81.5135	0.378719
14	newly 3 weeks spec	2684.	1581.42	{70., 1077.}	117.352	1.03454	newly 3 weeks cumul	12 513.	7250.99	{283., 5264.}	113.853	0.984116	newly 5 weeks spec	929.	488.11	{21., 628.}	102.305	0.966978	newly 5 weeks cumul	15 065.	8593.9	{386., 6733.}	112.262	0.970027	newly 57 weeks cumul	16 544.	9883.3	{611., 7638.}	110.998	0.923341
15	newly 3 weeks spec	3944.	1282.58	{0., 2025.}	91.1775	1.61211	newly 3 weeks cumul	16 141.	12 067.9	{3177., 17 109.}	90.975	0.583355	newly 5 weeks spec	1651.	274.37	{0., 324.}	94.3895	1.76068	newly 5 weeks cumul	20 293.	12 937.7	{3330., 18 078.}	89.4837	0.638406	newly 54 weeks cumul	22 727.	13 194.4	{3350., 18 248.}	89.0372	0.671111
16	newly 3 weeks spec	2270.	1019.34	{69., 1192.}	95.163	0.893738	newly 3 weeks cumul	7578.	3282.04	{463., 2512.}	93.3537	0.847698	newly 5 weeks spec	1786.	971.43	{15., 2328.}	94.0711	0.9599	newly 5 weeks cumul	11 349.	5231.62	{524., 6673.}	91.6567	0.838745	newly 50 weeks cumul	16 325.	20 011.5	{524., 85 263.}	144.022	0.855828
17	newly 3 weeks spec	4099.	2412.32	{100., 10 389.}	112.966	1.03271	newly 3 weeks cumul	15 754.	13 493.6	{999., 38 836.}	111.01	0.692503	newly 5 weeks spec	1851.	653.58	{14., 887.}	101.325	1.38226	newly 5 weeks cumul	20 371.	15 361.7	{1045., 62 533.}	108.902	0.752167	newly 56 weeks cumul	22 951.	16 411.2	{1105., 78 492.}	107.928	0.778326
18	newly 3 weeks spec	3126.	698.38	{0., 926.}	97.0793	2.2285	newly 3 weeks cumul	17 638.	8419.69	{1073., 16 537.}	98.1289	0.87947	newly 5 weeks spec	665.	164.	{0., 137.}	105.185	2.13374	newly 5 weeks cumul	19 872.	8894.85	{1073., 24 242.}	97.2814	0.907039	newly 60 weeks cumul	20 225.	9112.93	{1073., 24 256.}	97.6935	0.909348
19	newly 3 weeks spec	3015.	825.82	{122., 1256.}	93.9012	1.08494	newly 3 weeks cumul	11 397.	3474.07	{826., 4357.}	89.225	0.907758	newly 5 weeks spec	1523.	374.61	{14., 628.}	90.3473	1.25436	newly 5 weeks cumul	15 147.	4410.4	{1115., 7976.}	88.5007	0.932914	newly 51 weeks cumul	18 357.	5663.33	{1334., 21 889.}	83.9168	0.905098
20	newly 3 weeks spec	3891.	531.16	{0., 520.}	96.5269	2.31321	newly 3 weeks cumul	17 885.	6087.85	{127., 9434.}	92.9058	1.06357	newly 5 weeks spec	1246.	200.3	{0., 131.}	104.562	2.50901	newly 5 weeks cumul	21 430.	6599.26	{132., 9477.}	92.3764	1.1155	newly 60 weeks cumul	22 520.	6965.61	{132., 9477.}	93.1771	1.13073
21	newly 3 weeks spec	3638.	596.53	{0., 813.}	97.6583	2.29663	newly 3 weeks cumul	16 322.	6218.26	{64., 10 545.}	93.6467	1.08375	newly 5 weeks spec	1041.	162.98	{0., 96.}	101.356	2.36085	newly 5 weeks cumul	19 258.	6681.29	{64., 10 578.}	93.0005	1.12761	newly 60 weeks cumul	20 284.	6931.63	{64., 10 579.}	93.364	1.1434
22	newly 3 weeks spec	3362.	2025.69	{6., 1740.}	123.424	1.36141	newly 3 weeks cumul	10 760.	5255.75	{69., 9671.}	108.747	1.15792	newly 5 weeks spec	2166.	1372.06	{1., 1671.}	116.446	1.48378	newly 5 weeks cumul	15 662.	8614.7	{92., 11 290.}	113.851	1.17617	newly 50 weeks cumul	22 682.	18 552.5	{115., 82 688.}	121.4	1.12759
23	newly 3 weeks spec	3951.	991.3	{0., 1543.}	89.2771	1.9326	newly 3 weeks cumul	16 287.	15 093.9	{4439., 22 912.}	74.782	0.360711	newly 5 weeks spec	1653.	496.95	{0., 1050.}	94.3624	2.43099	newly 5 weeks cumul	20 536.	16 249.8	{5699., 32 105.}	74.837	0.396815	newly 55 weeks cumul	22 473.	18 189.2	{6507., 32 514.}	73.3768	0.374708
24	newly 3 weeks spec	2779.	618.32	{21., 301.}	105.036	1.50716	newly 3 weeks cumul	13 850.	3265.41	{388., 3471.}	94.1663	1.11174	newly 5 weeks spec	702.	268.06	{0., 116.}	119.769	1.77497	newly 5 weeks cumul	16 098.	3949.56	{443., 3991.}	96.4015	1.13935	newly 59 weeks cumul	16 529.	4359.13	{462., 4303.}	97.8948	1.13364
25	newly 3 weeks spec	3462.	893.91	{39., 1269.}	91.7282	1.15113	newly 3 weeks cumul	12 350.	2749.75	{135., 2557.}	89.8406	1.12412	newly 5 weeks spec	2037.	885.71	{12., 2463.}	89.7982	1.11047	newly 5 weeks cumul	17 226.	4523.25	{187., 6941.}	88.8191	1.08848	newly 50 weeks cumul	22 743.	18 402.7	{338., 85 251.}	117.144	1.01626
26	newly 3 weeks spec	3048.	1129.73	{1., 1138.}	105.621	1.5191	newly 3 weeks cumul	12 655.	5086.31	{173., 4507.}	103.954	1.04357	newly 5 weeks spec	1843.	551.65	{0., 523.}	102.983	1.8376	newly 5 weeks cumul	16 877.	6428.42	{212., 5985.}	103.193	1.09206	newly 52 weeks cumul	20 285.	7732.39	{246., 7491.}	102.519	1.11319
27	newly 3 weeks spec	3519.	880.06	{71., 1343.}	94.32	1.15013	newly 3 weeks cumul	12 256.	2771.66	{257., 2800.}	92.0326	1.109	newly 5 weeks spec	1833.	810.85	{26., 2469.}	91.1626	1.09499	newly 5 weeks cumul	16 564.	4413.73	{403., 7457.}	90.7123	1.05944	newly 50 weeks cumul	21 086.	19 607.2	{706., 85 290.}	119.657	0.950288
28	newly 3 weeks spec	2682.	812.32	{105., 575.}	100.072	1.07094	newly 3 weeks cumul	8965.	3146.85	{648., 2404.}	92.115	0.872082	newly 5 weeks spec	1792.	320.56	{14., 364.}	93.2701	1.34024	newly 5 weeks cumul	12 944.	4007.24	{897., 2997.}	91.4833	0.920342	newly 51 weeks cumul	19 275.	4785.23	{1192., 7670.}	87.9291	0.950112
29	newly 3 weeks spec	2188.	774.58	{26., 866.}	100.902	1.11142	newly 3 weeks cumul	8468.	5937.65	{147., 5537.}	116.246	0.871626	newly 5 weeks spec	1093.	286.35	{7., 233.}	104.647	1.47058	newly 5 weeks cumul	11 210.	6693.26	{188., 6153.}	112.045	0.927478	newly 53 weeks cumul	14 641.	7102.17	{215., 6351.}	107.676	1.00247
30	newly 3 weeks spec	3476.	945.02	{0., 1913.}	84.515	1.73425	newly 3 weeks cumul	15 376.	15 332.9	{4383., 29 391.}	77.9854	0.345017	newly 5 weeks spec	1413.	557.95	{0., 1488.}	92.2909	2.38947	newly 5 weeks cumul	19 115.	16 572.2	{5478., 34 164.}	73.9318	0.365247	newly 55 weeks cumul	20 923.	19 024.8	{6284., 34 164.}	70.2259	0.338526
31	newly 3 weeks spec	2766.	868.65	{1., 761.}	102.389	1.48998	newly 3 weeks cumul	10 733.	3992.32	{150., 2995.}	101.868	1.04585	newly 5 weeks spec	1803.	453.24	{0., 382.}	101.777	1.86809	newly 5 weeks cumul	14 870.	5066.19	{151., 3870.}	100.818	1.10983	newly 52 weeks cumul	18 960.	6394.37	{151., 6844.}	99.9611	1.14958
32	newly 3 weeks spec	2860.	1522.12	{10., 1222.}	116.657	1.28593	newly 3 weeks cumul	9853.	3442.95	{175., 7127.}	96.8774	1.12863	newly 5 weeks spec	1902.	1188.53	{1., 2055.}	112.358	1.42358	newly 5 weeks cumul	14 117.	6176.01	{201., 8260.}	103.28	1.12261	newly 50 weeks cumul	21 462.	19 441.6	{258., 84 774.}	115.463	0.993905
33	newly 3 weeks spec	1762.	523.56	{0., 404.}	103.757	2.01955	newly 3 weeks cumul	12 781.	5663.74	{77., 8058.}	97.6152	0.974768	newly 5 weeks spec	298.	200.29	{0., 122.}	141.158	1.88904	newly 5 weeks cumul	13 856.	6175.1	{77., 8058.}	99.1057	0.993187	newly 60 weeks cumul	13 996.	6525.52	{77., 8058.}	100.924	0.994056
34	newly 3 weeks spec	2684.	2317.18	{76., 2052.}	122.288	0.803814	newly 3 weeks cumul	12 876.	13 157.	{1159., 11 798.}	110.234	0.551277	newly 5 weeks spec	799.	603.34	{6., 947.}	112.678	1.02901	newly 5 weeks cumul	15 119.	14 895.1	{1183., 12 347.}	110.508	0.570832	newly 56 weeks cumul	15 839.	16 289.7	{1334., 63 449.}	112.715	0.568977
35	newly 3 weeks spec	685.	404.32	{0., 392.}	114.359	1.83744	newly 3 weeks cumul	10 337.	8465.53	{614., 9552.}	117.345	0.722978	newly 5 weeks spec	47.	154.	{0., 84.}	356.809	1.35271	newly 5 weeks cumul	10 552.	8845.07	{655., 9552.}	119.083	0.721113	newly 60 weeks cumul	10 552.	9118.88	{664., 9552.}	121.322	0.719491
36	newly 3 weeks spec	2872.	704.36	{0., 901.}	96.7423	2.06798	newly 3 weeks cumul	12 863.	8028.65	{86., 21 749.}	103.555	0.980175	newly 5 weeks spec	910.	184.18	{0., 190.}	103.242	2.26722	newly 5 weeks cumul	15 281.	8548.34	{86., 23 978.}	100.869	1.02368	newly 60 weeks cumul	16 833.	8830.6	{86., 23 987.}	99.4911	1.05031
37	newly 3 weeks spec	3362.	860.76	{46., 1476.}	97.2522	1.24381	newly 3 weeks cumul	13 277.	7078.37	{498., 7061.}	105.821	0.921014	newly 5 weeks spec	1507.	283.94	{4., 224.}	102.818	1.69415	newly 5 weeks cumul	17 055.	7849.94	{556., 8407.}	102.482	0.968819	newly 53 weeks cumul	19 554.	8268.49	{569., 9632.}	101.212	0.997147
38	newly 3 weeks spec	2972.	2571.77	{0., 4489.}	149.51	2.28872	newly 3 weeks cumul	16 469.	10 378.4	{219., 48 939.}	107.518	1.0857	newly 5 weeks spec	612.	832.1	{0., 1397.}	199.627	2.16327	newly 5 weeks cumul	18 339.	12 921.3	{235., 56 927.}	114.441	1.10837	newly 60 weeks cumul	18 646.	13 874.3	{235., 57 080.}	118.219	1.11432
39	newly 3 weeks spec	2387.	1733.36	{100., 1505.}	119.372	0.864528	newly 3 weeks cumul	10 499.	7279.84	{627., 5101.}	112.956	0.777495	newly 5 weeks spec	937.	601.59	{8., 967.}	98.0309	0.870839	newly 5 weeks cumul	12 897.	8885.82	{715., 7080.}	111.321	0.772494	newly 57 weeks cumul	14 107.	10 568.	{837., 12 337.}	108.579	0.71711
40	newly 3 weeks spec	2220.	612.88	{6., 315.}	108.297	1.43914	newly 3 weeks cumul	13 427.	2858.98	{187., 2153.}	95.1155	1.10713	newly 5 weeks spec	616.	274.83	{0., 133.}	123.758	1.76778	newly 5 weeks cumul	15 217.	3558.59	{188., 2350.}	97.934	1.12665	newly 59 weeks cumul	15 737.	3976.33	{188., 2350.}	99.5061	1.12418
41	newly 3 weeks spec	3803.	591.34	{0., 881.}	97.4625	2.32371	newly 3 weeks cumul	18 778.	6953.71	{1133., 14 843.}	90.3231	0.889418	newly 5 weeks spec	894.	155.63	{0., 129.}	103.654	2.27773	newly 5 weeks cumul	21 558.	7399.98	{1133., 15 243.}	90.422	0.925639	newly 60 weeks cumul	22 565.	7601.6	{1133., 15 302.}	90.8284	0.939953
42	newly 3 weeks spec	3308.	1106.98	{31., 1761.}	97.2963	1.13914	newly 3 weeks cumul	14 867.	5952.53	{110., 7651.}	102.186	1.09744	newly 5 weeks spec	1090.	336.1	{15., 900.}	81.8257	1.0297	newly 5 weeks cumul	17 792.	6861.51	{147., 9795.}	99.3012	1.07393	newly 57 weeks cumul	18 900.	7858.07	{256., 12 657.}	95.9354	1.00068
43	newly 3 weeks spec	4665.	608.06	{0., 875.}	96.0656	2.32791	newly 3 weeks cumul	19 701.	7273.2	{1160., 18 038.}	90.1208	0.916164	newly 5 weeks spec	1372.	161.28	{0., 143.}	100.442	2.41901	newly 5 weeks cumul	23 754.	7732.07	{1203., 19 200.}	89.3562	0.96747	newly 60 weeks cumul	25 089.	7959.38	{1216., 19 260.}	89.7585	0.984754
44	newly 3 weeks spec	3246.	896.2	{36., 611.}	103.2	1.37969	newly 3 weeks cumul	17 555.	6206.93	{406., 3419.}	103.085	1.12999	newly 5 weeks spec	782.	248.31	{1., 279.}	106.421	1.58749	newly 5 weeks cumul	19 996.	6896.74	{452., 4051.}	102.823	1.14626	newly 59 weeks cumul	20 762.	7258.72	{463., 4478.}	103.09	1.14668
45	newly 3 weeks spec	3565.	969.14	{0., 1567.}	90.4454	2.00504	newly 3 weeks cumul	13 069.	14 991.6	{3484., 18 755.}	89.2881	0.380334	newly 5 weeks spec	1772.	455.39	{0., 929.}	95.741	2.46143	newly 5 weeks cumul	17 480.	16 080.6	{3790., 26 339.}	80.8132	0.407789	newly 55 weeks cumul	20 564.	17 823.7	{5635., 36 243.}	79.0755	0.40779
46	newly 3 weeks spec	4749.	2422.88	{149., 9679.}	105.325	1.05001	newly 3 weeks cumul	18 136.	15 218.9	{1064., 54 193.}	114.243	0.751306	newly 5 weeks spec	1998.	642.92	{13., 900.}	99.6657	1.40681	newly 5 weeks cumul	23 379.	17 053.4	{1297., 62 979.}	109.984	0.803929	newly 56 weeks cumul	26 044.	18 095.4	{1461., 94 179.}	108.499	0.8203
47	newly 3 weeks spec	3376.	2465.48	{57., 10 478.}	121.703	1.03544	newly 3 weeks cumul	16 086.	14 247.4	{580., 53 389.}	114.057	0.76849	newly 5 weeks spec	1272.	600.13	{4., 897.}	107.29	1.33877	newly 5 weeks cumul	19 497.	16 027.4	{621., 64 880.}	113.063	0.79995	newly 56 weeks cumul	20 865.	17 397.4	{698., 66 429.}	113.986	0.798877
48	newly 3 weeks spec	3671.	395.53	{35., 718.}	89.2256	1.25803	newly 3 weeks cumul	14 471.	1873.12	{251., 3401.}	88.1531	1.10416	newly 5 weeks spec	1686.	220.16	{6., 444.}	87.5813	1.38651	newly 5 weeks cumul	18 539.	2389.13	{307., 4734.}	87.5475	1.11544	newly 51 weeks cumul	21 237.	3281.8	{352., 6843.}	84.6728	1.06385
49	newly 3 weeks spec	2539.	1397.6	{118., 1808.}	100.121	0.775207	newly 3 weeks cumul	11 374.	6601.35	{1508., 6793.}	102.778	0.723367	newly 5 weeks spec	806.	595.8	{9., 1079.}	94.8065	0.710315	newly 5 weeks cumul	13 685.	8075.24	{1646., 8792.}	100.901	0.705256	newly 57 weeks cumul	14 608.	10 054.4	{1844., 13 315.}	99.7483	0.638573
50	newly 3 weeks spec	2519.	671.08	{45., 1626.}	82.7749	1.06773	newly 3 weeks cumul	12 653.	4541.14	{384., 8211.}	85.6682	0.883111	newly 5 weeks spec	744.	266.37	{2., 573.}	82.4637	1.16921	newly 5 weeks cumul	14 778.	5209.03	{500., 8595.}	84.8503	0.87494	newly 58 weeks cumul	15 627.	5988.73	{595., 10 887.}	84.7456	0.838001
